# Pathogenic Crosstalk Between the Peripheral and Central Nervous System in Rheumatic Diseases: Emerging Evidence and Clinical Implications

**DOI:** 10.3390/ijms26136036

**Published:** 2025-06-24

**Authors:** Marino Paroli, Maria Isabella Sirinian

**Affiliations:** Center for Allergology and Immunology, Department of Clinical, Internal, Anesthesiologic and Cardiovascular Sciences, Sapienza University of Rome, Polo Pontino, 04100 Latina, Italy; mariaisabellasirinian@gmail.com

**Keywords:** neuroimmune dysregulation, glial activation, small-fiber neuropathy, cytokines, neuromodulators

## Abstract

Systemic autoimmune rheumatic diseases (SARDs), such as rheumatoid arthritis (RA), systemic lupus erythematosus (SLE), and Sjögren’s syndrome (SS), are traditionally characterized by chronic inflammation and immune-mediated damage to joints and other tissues. However, many patients also experience symptoms such as widespread pain, persistent fatigue, cognitive dysfunction, and autonomic disturbances that cannot be attributed directly or entirely to peripheral inflammation or structural pathology. These conditions suggest the involvement of interactions between the nervous and immune systems, which probably include both peripheral and central components. This review summarizes the current knowledge of neurological and neuroimmune mechanisms that may contribute to these symptoms in SARDs. Glial cell activation and neuroinflammation within the central nervous system (CNS), small-fiber neuropathy (SFN) affecting peripheral nociceptive pathways, central pain sensitization, and autonomic nervous system dysfunction will be discussed. In addition, the role of molecular mediators, including cytokines, neuropeptides, and microRNAs, that could potentially modulate neuroimmune signaling will be highlighted. Integrating findings from pathology, immunology, and neuroscience, this review seeks to provide a useful framework for understanding neuroimmune dysregulation in SARDs. It also highlights the clinical relevance of these mechanisms and summarizes new directions for diagnosis and treatment.

## 1. Introduction

The clinical spectrum of systemic autoimmune rheumatic diseases (SARDs) goes beyond joint inflammation and organ damage. In recent years, neurological manifestations have been increasingly recognized as key components of these diseases, both in terms of disease burden and diagnostic complexity [1,2,3].

Patients often report persistent symptoms such as widespread musculoskeletal pain, fatigue, cognitive impairment, dysesthesia, and signs of autonomic dysfunction. Although traditionally considered secondary or nonspecific, these manifestations are now recognized as potentially arising from distinct pathophysiologic mechanisms involving the nervous system [4,5,6,7].

Abundant evidence suggests that the nervous and immune systems are closely linked through bidirectional communication networks involving cytokines, neuropeptides, glial cells, and sensory/autonomic neurons [8,9,10]. This neuroimmune interaction may contribute to low-grade local and systemic inflammation, which may help explain the disproportionate clinical features observed in patients with SARDs compared with actual peripheral tissue damage. For example, the presence of small-fiber neuropathy, central sensitization, or neuroinflammation may underlie persistent pain and fatigue even in patients with well-controlled synovitis [11,12].

Despite this knowledge, the pathogenetic basis of neuroimmune dysfunction in SARDs remains fragmentary, and a unifying framework is still lacking.

The aim of this review is to provide a summary of the mechanisms underlying neuroimmune dysregulation in SARDs, with a focus on the interaction between the peripheral and central nervous systems. The most important pathological processes, including glial activation, small-fiber neuropathy, central sensitization, and autonomic dysfunction, will be discussed, along with the role of molecular mediators that modulate these interactions.

By integrating findings from rheumatology, immunology, and neurobiology, it will be possible in the future to elucidate how these processes contribute to the complex clinical presentation of SARDs and to delineate implications for the diagnosis and treatment of nervous system-mediated manifestations.

## 2. The Neuroimmune Interface in Rheumatic Diseases

The immune and nervous systems are closely interconnected and share not only the same molecular messengers, such as cytokines and chemokines [13,14,15], but also structural and functional interfaces that allow bidirectional communication [16,17]. This relationship is particularly relevant in SARDs, where immune dysregulation is systemic and the clinical presentation often includes neurological or neurobehavioral symptoms [18,19,20]. Peripherally, immune cells such as macrophages, mast cells, and T lymphocytes interact with nociceptive sensory neurons through cytokines (such as IL-1β, TNF-α, and IL-17) and neuropeptides such as substance P and calcitonin gene-related peptide (CGRP) [21,22,23,24,25,26]. This interaction can lead to peripheral sensitization, in which normally harmless stimuli become pathologically painful. In this regard, damage to unmyelinated C-fibers and thinly myelinated Aδ-fibers, characteristic of small-fiber neuropathy (SFN), has been documented in several SARDs, suggesting direct neuroimmune damage to peripheral nerves [27,28,29,30].

Centrally, inflammatory mediators can access the brain through permeable regions of the blood–brain barrier (BBB) via active transport mechanisms or through circumventricular organs [31,32,33]. Once inside the central nervous system (CNS), these mediators can activate resident glial cells, such as astrocytes and microglia, leading to a prolonged state of neuroinflammation. This process has been implicated in both central sensitization and the development of fatigue and mood disorders [34,35,36,37]. Importantly, the integrity of the blood–brain barrier and blood–nerve barrier can be compromised in SARDs, thus facilitating the entry of peripheral immune cells or autoantibodies into normally immune-privileged sites [38,39,40]. Recent studies have identified perivascular immune cell infiltration, increased glial reactivity, and upregulation of inflammatory markers in the spinal cord and brain tissues of animal models of arthritis and lupus, supporting this mechanism [23,41,42].

Overall, the results obtained from various experimental studies suggest that the nervous system is not simply a passive receptor of immune signals but plays an active role in shaping the inflammatory response and its systemic consequences. The concept of a dynamic neuroimmune interface thus provides a framework for understanding how diseases of immunologic origin can produce symptoms of neurologic origin, even in the absence of structural lesions detectable by conventional imaging techniques.

## 3. Neuroinflammation and Glial Activation

Neuroinflammation, defined as the activation of immune responses within the CNS, has thus emerged as a key factor in the neurological and systemic manifestations observed in SARDs. Unlike classic CNS inflammation associated with neurodegenerative or infectious diseases, neuroinflammation in SARDS is often more subtle, more widespread, and chronic. The various studies to date suggest that it is mediated predominantly by resident glial cells, microglia, and astrocytes, which respond to systemic immune signals and contribute to altered neural processing even in the absence of obvious CNS lesions [43,44].

Microglia, the main innate immune cells of the CNS, are extremely sensitive to peripheral inflammatory stimuli. In animal models of systemic autoimmune diseases such as lupus and arthritis, peripheral immune activation leads to microglial priming and subsequent production of pro-inflammatory cytokines within the CNS [45,46,47,48,49]. These cytokines can alter synaptic transmission, promote excitotoxicity, and impair neural network stability. Astrocytes, traditionally considered support cells, also play an active immunomodulatory role by producing chemokines, modulating glutamate reuptake, and affecting blood–brain barrier (BBB) permeability [50,51,52].

Evidence from preclinical models also indicates that systemic inflammation can lead to long-term changes in glial function, even after resolution of the initial peripheral immune response [53]. In mouse models of collagen-induced arthritis and systemic lupus erythematosus, for example, increased glial reactivity was observed in the hippocampus, cortex, and spinal cord, regions involved in pain perception, memory, and mood regulation. These findings are consistent with clinical reports of cognitive dysfunction and mood disorders in patients with SARDs [47,54,55]. In humans, neuroimaging studies have provided indirect support for glial activation.

Positron emission tomography (PET) imaging using ligands that bind to translocator protein (TSPO), a marker of microglial activation, has shown increased uptake in patients with chronic pain and fatigue, including those with fibromyalgia and systemic autoimmune diseases [56,57,58,59,60].

Although data on populations with SARDs are still limited, these findings suggest that low-grade neuroinflammation may be an underreported but clinically relevant phenomenon. Specifically, glial activation may support a vicious cycle by amplifying nociceptive signaling and increasing central sensitization, contributing to the persistence of pain and fatigue even in the absence of active peripheral inflammation.

**Figure 1:**
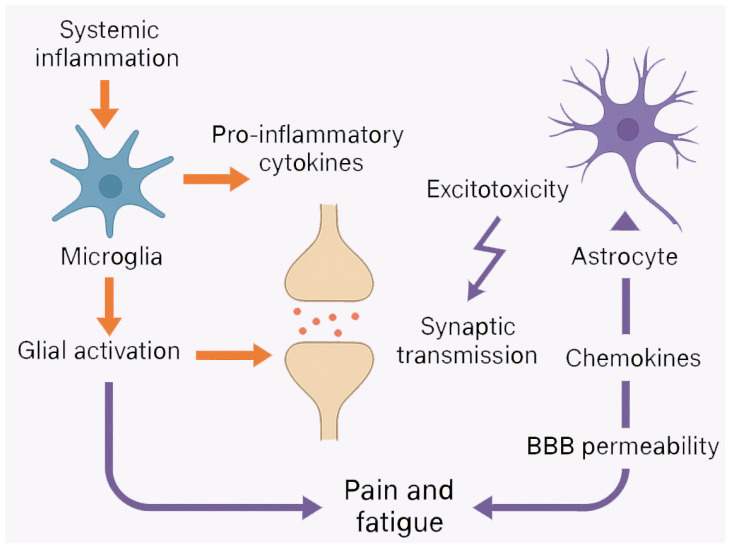
Neuroinflammation and glial activation in systemic autoimmune rheumatic diseases (SARDs). This diagram illustrates how systemic inflammation activates microglia and astrocytes, triggering the release of pro-inflammatory cytokines, synaptic disruption, excitotoxicity, and increased blood–brain barrier permeability. These processes converge in sustaining pain and fatigue through the glial–cytokine–pain axis.

This “glial–cytokine–pain” axis also represents a potential therapeutic target, and several preclinical studies have shown that pharmacological inhibition of glial activation can attenuate pain behaviors in autoimmune models [61,62,63] (Figure 1).

## 4. Small-Fiber Neuropathy and Peripheral Nerve Damage

Small-fiber neuropathy (SFN) is a form of peripheral nerve damage that selectively affects fine myelinated Aδ fibers and unmyelinated C fibers, which are mainly responsible for pain, thermal sensation, and autonomic regulation [64,65]. Although traditionally under-recognized, SFN is increasingly being reported in SARDs, including systemic lupus erythematosus (SLE), Sjögren’s syndrome, rheumatoid arthritis (RA), and sarcoidosis [66,67,68,69]. Clinical presentation may include burning pain, allodynia, paresthesia, dysautonomia, and hyperalgesia, symptoms that often overlap with fibromyalgia, leading to underdiagnosis or misclassification.

Histopathologically, SFN is characterized by reduced intraepidermal nerve fiber density (IENFD) on skin biopsies [70]. In several studies, a significant proportion of patients with SARDs and chronic widespread pain showed pathological confirmation of SFN, even in the absence of conventional large-fiber neuropathy on nerve conduction studies. This supports the hypothesis that a subset of patients with unexplained pain may, in fact, have objective evidence of peripheral nociceptive fiber damage [71].

The mechanisms that determine SFN in SARDs are multifactorial. Direct immune-mediated damage via autoantibodies or complement activation has been proposed, particularly in diseases with known autoantibody profiles, such as anti-Ro/SSA in Sjögren’s syndrome [72]. In addition, chronic systemic inflammation and elevated levels of pro-inflammatory cytokines may exert neurotoxic effects on small fibers. Ischemic damage due to microvascular involvement, commonly seen in systemic vasculitis or lupus, may also contribute to nerve fiber loss [73,74].

Importantly, SFN not only causes localized neuropathic symptoms but also contributes to systemic manifestations such as fatigue, orthostatic intolerance, and gastrointestinal dysmotility through its impact on autonomic fibers [75,76,77]. In this regard, SFN acts as a pathological link between peripheral inflammation and the expression of central symptoms, particularly when combined with central sensitization mechanisms.

The distinction between SFN and central pain mechanisms is clinically relevant but often confusing. Some patients with confirmed SFN also exhibit features of central sensitization, such as temporal summation of pain or altered conditional pain modulation. This overlap supports a model in which peripheral nerve injury may act as a trigger for maladaptive central plasticity, further amplifying pain signals [78,79].

**Figure 2:**
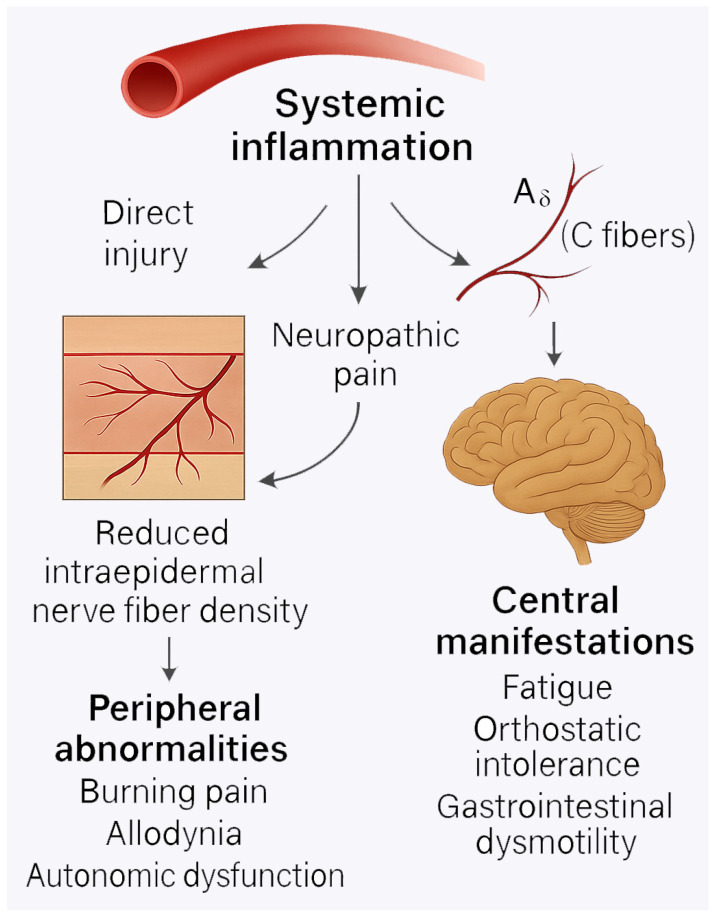
Small-fiber neuropathy and peripheral nerve damage in systemic autoimmune rheumatic diseases (SARDs) This diagram illustrates the reduction in intraepidermal nerve fiber density by systemic inflammation and its impact in SARDs. Systemic inflammation results in direct immune-mediated or ischemic injury of small nerve fibers, leading to central manifestations including fatigue, orthostatic intolerance, and gastrointestinal dysmotility.

Despite its significance, SFN remains underdiagnosed in clinical practice. Routine screening with skin biopsy, quantitative sensory testing, or corneal confocal microscopy is still not standard in rheumatology clinics. However, increased awareness and early recognition of SFN may have important therapeutic implications, as some treatments targeted for neuropathic pain differ from those used for inflammatory or nociplastic pain [34] (Figure 2).

## 5. Central Sensitization and Chronic Pain

Central sensitization refers to an increased responsiveness of nociceptive neurons in the CNS to normal or subthreshold afferent inputs [80]. It is a key mechanism underlying chronic pain states, particularly when pain persists in the absence of active peripheral inflammation or tissue damage. In SARDs, central sensitization is increasingly recognized as a major contributor to chronic pain and as a potential explanation for discrepancies between objective disease activity and subjective symptom severity [81,82,83,84].

In 2017, the International Association for the Study of Pain (IASP) introduced “nociplastic pain” as a third mechanistic descriptor of pain, alongside nociceptive and neuropathic pain, based on the recommendations of the IASP Terminology Task Force. Nociplastic pain is defined as “pain resulting from altered nociception, despite no clear evidence of actual or threatened tissue damage causing peripheral nociceptor activation or evidence of disease or injury of the somatosensory system causing the pain.” [85]. Clinical features of central sensitization include hyperalgesia (increased pain from normally painful stimuli), allodynia (pain from normally non-painful stimuli), and widespread distribution of pain [86,87].

These features are commonly observed in patients with rheumatoid arthritis, systemic lupus erythematosus, psoriatic arthritis, and spondyloarthritis, particularly in those who also meet criteria for fibromyalgia. The overlap between central sensitization and fibromyalgia has given rise to the concept of “secondary fibromyalgia,” in which centrally mediated pain amplification develops in the context of primary rheumatic disease [83,88,89,90,91,92].

From a mechanistic point of view, central sensitization involves dysfunctional pain modulation pathways at the spinal and supraspinal levels. Persistent nociceptive input from inflamed joints or damaged peripheral nerves can induce long-term potentiation in dorsal horn neurons, a process mediated by glutamate, substance P, and brain-derived neurotrophic factor (BDNF) [22,93,94,95]. These changes are believed to lower the pain transmission threshold and increase the excitability of central neurons.

Glial cells also play a central role in maintaining central sensitization. Activated microglia and astrocytes that release pro-inflammatory cytokines and neuroexcitatory mediators contribute to a neuroinflammatory environment within the spinal cord and brain [22,96]. These processes could be further amplified by impairment of descending inhibitory control by brainstem structures such as the periaqueductal gray and ventromedial rostral medulla, which normally suppress pain transmission [97,98,99].

Functional neuroimaging studies have provided insights into the neural correlates of central sensitization in patients with SARDs [100,101,102]. In patients with fibromyalgia and in subsets of patients with SARDs with chronic pain, altered activity and connectivity have been observed in brain regions involved in pain processing, such as the insula, anterior cingulate cortex, and thalamus. These results support the hypothesis that central amplification of pain is a measurable and biologically based phenomenon [103].

Importantly, central sensitization is associated with poor response to conventional anti-inflammatory or immunosuppressive therapies. This has led to the development of pain phenotyping approaches, which classify patients according to the dominant pain mechanism (inflammatory, neuropathic, or nociplastic) and guide treatment accordingly [91,104,105,106]. For patients with prominent central sensitization, pharmacological interventions targeting central neurotransmission, including serotonin–norepinephrine reuptake inhibitors, tricyclic antidepressants, and gabapentinoids, and nonpharmacological strategies such as cognitive-behavioral therapy, graded exercise, and neuromodulation are often more effective [107,108,109,110,111].

## 6. Dysregulation of the Autonomic Nervous System

The autonomic nervous system (ANS), which includes sympathetic and parasympathetic branches, plays a key role in maintaining homeostasis in the cardiovascular, gastrointestinal, thermoregulatory, and immune systems [112]. In SARDs, ANS dysfunction, commonly referred to as dysautonomia, has been increasingly recognized and is associated with a wide range of symptoms, including orthostatic intolerance, fatigue, palpitations, gastrointestinal dysmotility, and thermoregulatory disturbances [113,114,115]. These manifestations often contribute substantially to the burden of disease but remain underdiagnosed and poorly understood in clinical practice.

Numerous studies have documented altered autonomic function in patients with SARDs using objective tools such as heart rate variability (HRV) analysis, tilt-table tests, and sudomotor function assessment. Reduced HRV, indicating altered parasympathetic tone and sympathetic–vagal imbalance, is one of the most consistent findings in diseases such as rheumatoid arthritis, systemic lupus erythematosus, and Sjögren’s syndrome [116,117,118]. These alterations have been associated with increased systemic inflammation, disease activity, and fatigue.

The mechanisms that determine ANS dysregulation in SARDs are multifactorial and not yet fully elucidated. It is currently believed that inflammatory cytokines may directly affect autonomic centers in the brainstem, alter baroreceptor sensitivity, and modulate vagal afferent signaling. In addition, immune-mediated damage to small autonomic fibers, as seen in small-fiber neuropathy, can lead to regional or systemic autonomic symptoms, including postural orthostatic tachycardia syndrome (POTS), orthostatic hypotension, and gastrointestinal dysmotility [119].

The emerging concept of “inflammatory reflex,” mediated by the vagus nerve, offers an interesting framework for understanding neuroimmune interactions in this context. It has been shown that activation of efferent vagal pathways suppresses cytokine production through anti-inflammatory cholinergic signaling, a mechanism that may be impaired in patients with chronic inflammation and low parasympathetic tone. This bidirectional loop suggests that autonomic dysfunction may not only be a consequence of inflammation but also a potential amplifier of systemic immune activation [120,121,122,123].

In addition to contributing to systemic symptoms, autonomic imbalance can influence the course and severity of underlying rheumatic disease. Sympathetic overactivity has been implicated in endothelial dysfunction, increased cardiovascular risk, and impaired immune regulation, all of which are relevant to the long-term outcomes of SARDs [124]. In contrast, strategies aimed at improving vagal tone, such as biofeedback, neuromodulation, and exercise training, have shown promise in modulating both autonomic function and inflammatory responses [118,125].

Clinically, recognizing autonomic dysfunction is therefore critical for comprehensive symptom management. Patients with overt dysautonomia may benefit from targeted therapies, including beta-blockers, fludrocortisone, midodrine, or nonpharmacologic interventions, such as increased fluid and salt intake, compression garments, and physical rehabilitation. Differentiating these symptoms from primary disease activity may also prevent unnecessary escalation of immunosuppressive treatment.

## 7. Emerging Molecular Mediators

The interface between the nervous and immune systems in SARDs is orchestrated by a complex network of molecular mediators. These include classical pro-inflammatory cytokines, neuropeptides, growth factors, and, more recently, noncoding RNAs and neuronal receptors with immunomodulatory properties. Understanding the role of these mediators provides insight into the mechanisms underlying neuroimmune dysregulation and highlights potential therapeutic targets that transcend conventional anti-inflammatory strategies.

Pro-inflammatory cytokines, such as tumor necrosis factor-alpha (TNF-α), interleukin-6 (IL-6), and IL-17, are central in the pathogenesis of SARDs and also exert direct effects on neuronal and glial cells. TNF-α can sensitize nociceptors and promote central sensitization through its action on spinal cord neurons and glial cells [126,127]. IL-6 is known to cross the blood–brain barrier and alter neurotransmitter metabolism, contributing to fatigue, mood disorders, and pain amplification [128,129,130]. IL-17, in addition to its pro-inflammatory role in peripheral tissues, has been implicated in altering the integrity of the blood–brain and blood–brain barrier and promoting neuroinflammation [131,132,133].

Neuropeptides such as CGRP, substance P, and vasoactive intestinal peptide (VIP) are released from sensory neurons and immune cells and act as bidirectional messengers in neuroimmune communication. CGRP has vasodilatory and immunoregulatory functions and is elevated in chronic pain conditions [134,135]. It can also modulate T-cell responses and dendritic cell activity [136,137]. Substance P promotes leukocyte trafficking and may amplify neurogenic inflammation through its action on neurokinin-1 receptors [138]. The balance and spatial context of these neuropeptides influence whether neuroimmune interactions lead to protective or pathological outcomes.

Brain-derived neurotrophic factor (BDNF) and nerve growth factor (NGF) are neurotrophins involved in neuronal survival and plasticity, but they are also upregulated in chronic inflammation and pain. NGF, in particular, is a potent nociceptor sensitizer and has been linked to both peripheral and central sensitization. Therapies targeting NGF (e.g., anti-NGF monoclonal antibodies) have shown promise in osteoarthritis and may have applications in SARDs with prominent pain syndromes [139,140,141,142].

MicroRNAs (miRNAs), small noncoding RNAs that regulate gene expression at the post-transcriptional level, have emerged as key regulators of immune responses and neuronal function. Dysregulated expression of miRNAs such as miR-146a, miR-155, and miR-124 has been reported in SARDs and is associated with both systemic inflammation and neuroinflammatory signaling. These molecules may serve as biomarkers of disease activity or response to treatment and represent potential therapeutic targets through RNA-based approaches [143,144,145].

**Figure 3:**
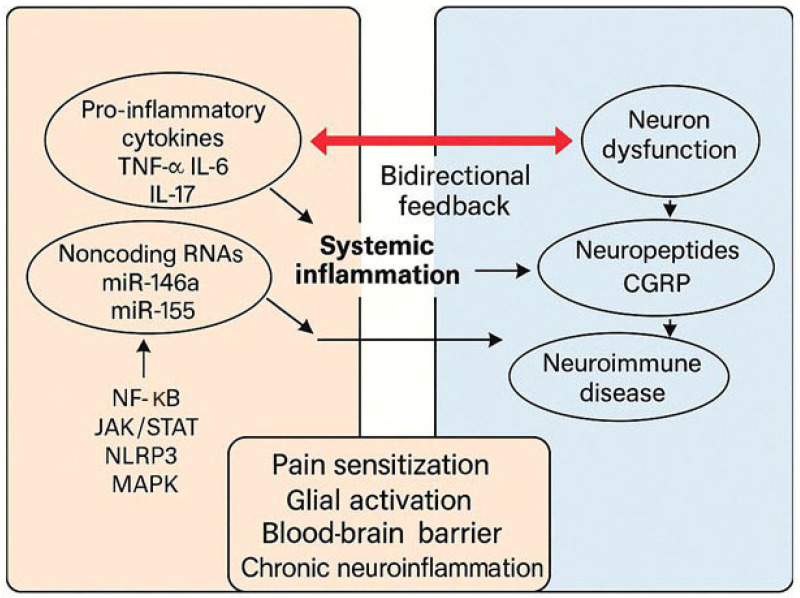
Systemic inflammation during SARDs is bidirectionally linked to neuroinflammation.

Receptors and signaling pathways at the neuroimmune interface, such as toll-like receptors (TLRs), purinergic receptors (e.g., P2X7), and the endocannabinoid system, also contribute to neuroinflammatory responses. TLRs on glial cells can be activated by damage-associated molecular patterns (DAMPs) released during systemic inflammation [146]. Interestingly, their signaling pathway has recently been linked to nociceptive pain in fibromyalgia involving microglia activation and M1/M2 polarization [147], while purinergic signaling modulates pain and cytokine release. In this regard, it has been shown that P2X7 receptor activation plays a key role in pain development by promoting the release of pro-inflammatory cytokines. Expressed in both the nervous and immune systems, it contributes to neuroimmune regulation and pain signaling. ATP triggers P2X7, opening non-selective cation channels and activating inflammatory pathways. The receptor’s involvement in pain and inflammation has been well established [148]. Modulation of these pathways may therefore provide new strategies to counter chronic pain and neuroimmune imbalance [149] (Figure 3).

This diagram illustrates some of the molecular mediators linking systemic inflammation and neuroimmune dysfunction in SARDs. It highlights the interaction between pro-inflammatory cytokines, neuropeptides, and non-coding RNA in the induction of glial activation, neuroimmune dysfunction, and ultimately the onset of pain.

## 8. Clinical Implications

As mentioned in the previous paragraphs, the recognition of neuroimmune dysregulation as a central component of SARDs has important clinical implications that extend to diagnosis, patient stratification, and therapeutic decision-making. Symptoms such as chronic pain, fatigue, cognitive dysfunction, and autonomic disturbances are frequently reported by patients with SARDs, yet they are often underestimated in routine clinical evaluations, which focus primarily on joint inflammation or serologic activity. Understanding the contribution of central and peripheral neuroimmune mechanisms offers a new lens through which to interpret and manage these symptoms.

In this regard it should be emphasized that autoimmune and inflammatory conditions can affect the central nervous system, leading to specific disorders such as myelitis. Advances in imaging and the identification of neural autoantibodies have significantly enhanced the diagnosis of immune-mediated myelopathies. Early recognition and targeted immunotherapy are essential to prevent long-term disability, making comprehensive diagnostic evaluation crucial in all cases of acute or subacute myelopathy [150].

Of particular relevance, due to its high morbidity, is neuropsychiatric systemic lupus erythematosus (NPSLE), a severe manifestation of SLE that affects the nervous system, with a complex and multifactorial pathogenesis. Factors contributing to its onset include genetic predisposition, alteration of the blood–brain barrier, vascular damage, autoantibodies, cytokines, and neuronal damage. Clinical heterogeneity and overlapping symptoms with other neuropsychiatric disorders represent a clinical challenge and complicate diagnosis [151].

One of the most significant implications is the dissociation between inflammation and symptom severity in many patients. Individuals may experience high levels of pain or fatigue despite quiescent peripheral inflammation, leading to frustration, misclassification, or inappropriate intensification of immunosuppressive therapy. In contrast, some patients with active inflammation may not report substantial symptoms, underscoring the heterogeneity of neuroimmune responses. Based on available data, it could be hypothesized that, in chronic inflammatory rheumatic diseases, persistent nociceptive input from inflamed joints may trigger a process of central sensitization. This phenomenon would lead to abnormal amplification of pain signaling within the central nervous system, resulting in increased pain perception even in areas not directly affected by inflammation. As a result, patients may experience clinical symptoms similar to those of primary fibromyalgia, such as widespread pain, fatigue, and sleep disturbances. However, the exact molecular mechanisms involved in this process remain unclear and require further investigation.

Integrating validated tools for assessing central sensitization, neuropathic pain characteristics, and autonomic dysfunction, such as the Central Sensitization Inventory or COMPASS-31, can help clinicians identify noninflammatory factors contributing to the symptom burden [152,153].

The recognition of distinct pain phenotypes, such as inflammatory, neuropathic, and nociplastic pain, is increasingly desired in rheumatology. This approach supports a more personalized treatment strategy. For example, patients with central sensitization may benefit more from neuromodulatory agents (e.g., duloxetine, amitriptyline, pregabalin) or nonpharmacologic interventions (e.g., graded exercise therapy, cognitive-behavioral therapy) rather than increased disease-modifying antirheumatic drugs (DMARDs). Similarly, small-fiber neuropathy may respond to specific treatments for neuropathic pain or, in selected cases, to intravenous immunoglobulins [154].

The presence of dysautonomia also warrants targeted management. Interventions such as hydration, compression garments, saline supplementation, and pharmacological modulation can substantially improve the quality of life of affected patients. In addition, some evidence suggests that enhancement of vagal tone through exercise, breathing techniques, or vagus nerve stimulation may have immunomodulatory effects and improve both autonomic and inflammatory outcomes [155].

In addition to guiding symptom-specific therapy, neuroimmune markers may offer future value in disease monitoring and prognosis. Molecular signatures involving cytokines, neuropeptides, or microRNAs could serve as biomarkers to identify patients at risk for chronic pain or fatigue syndromes and help predict response to treatment. Functional neuroimaging and quantitative sensory testing, although currently limited to research, could contribute to clinical stratification.

Importantly, these insights support the need for a more multidisciplinary approach to SARDs’ care. Collaboration among rheumatologists, neurologists, pain specialists, and psychologists may be essential to adequately address the complex and multifactorial symptomatology experienced by patients. Recognizing and validating these symptoms not as psychological or secondary disorders but as biologically based phenomena can improve patient engagement, satisfaction, and outcomes.

In summary, understanding the neuroimmune basis of pain, fatigue, and autonomic symptoms in SARDs challenges traditional paradigms of disease assessment and expands the range of therapeutic tools. Integrating this knowledge into clinical practice is essential to provide more comprehensive, compassionate, and effective care.

## 9. Conclusions and Future Directions

SARDs present with a broad spectrum of symptoms that go far beyond joint inflammation and organ involvement. Chronic pain, fatigue, cognitive dysfunction, and autonomic disturbances are common and debilitating features that are not fully explained by peripheral immune activity. The growing body of evidence on neuroimmune interactions ranging from small-fiber neuropathy to central sensitization, glial activation, and autonomic dysregulation underscores the need for a more integrated pathophysiologic framework in rheumatology [156].

This review highlights how the nervous system is not simply a passive victim of systemic inflammation but an active participant in disease expression and persistence. Peripheral and central mechanisms interact dynamically, with molecular mediators such as cytokines, neuropeptides, and microRNAs shaping the crosstalk between immune cells and neurons. These processes help explain the often-observed discrepancy between objective inflammatory markers and subjective symptom burden in SARDs.

Clinically, this understanding invites a paradigm shift in the way symptoms are assessed and managed. It calls for the incorporation of pain phenotyping, assessment of autonomic function, and neuroimmune biomarkers into clinical workflows. From a therapeutic perspective, it supports a more personalized and multimodal approach that combines immunosuppression with neuromodulatory and rehabilitative strategies, depending on the dominant symptoms.

Despite these advances, many questions remain. The precise mechanisms linking immune activation to neural dysfunction are still incompletely understood. Prospective studies integrating neuroimaging, electrophysiological data, and molecular profiles are needed to better characterize neuroimmune phenotypes in SARDs. In addition, clinical trials testing interventions that specifically target neuroimmune pathways, such as vagus nerve stimulation, anti-neurotrophin therapies, or RNA-based approaches, are still in their infancy. Future research should also aim to validate accessible biomarkers that can guide diagnosis and treatment stratification.

## References

[1] Mease P.J. (2024). Navigating the complexity of pain in psoriatic arthritis and axial spondyloarthritis. Curr. Opin. Rheumatol..

[2] Murphy A.E., Minhas D., Clauw D.J., Lee Y.C. (2023). Identification and management of nociplastic pain in individuals with rheumatic diseases: A Narrative Review. Arthritis Care Res..

[3] Lacagnina M.J., Heijnen C.J., Watkins L.R., Grace P.M. (2021). Autoimmune regulation of chronic pain. PAIN Rep..

[4] Davies K., Dures E., Ng W.F. (2021). Fatigue in inflammatory rheumatic diseases: Current knowledge and areas for future research. Nat. Rev. Rheumatol..

[5] Thombs B.D., Adams C. (2022). Coping with fatigue in inflammatory rheumatic diseases. Lancet Rheumatol..

[6] Clauw D., Sarzi-Puttini P., Pellegrino G., Shoenfeld Y. (2024). Is fibromyalgia an autoimmune disorder?. Autoimmun. Rev..

[7] Myasoedova E., Sattui S.E., Lee J., O’Brien J.T., Makris U.E. (2024). Cognitive impairment in individuals with rheumatic diseases: The role of systemic inflammation, immunomodulatory drugs, and comorbidities. Lancet Rheumatol..

[8] Linnerbauer M., Wheeler M.A., Quintana F.J. (2020). Astrocyte Crosstalk in CNS Inflammation. Neuron.

[9] Wu M., Song G., Li J., Song Z., Zhao B., Liang L., Li W., Hu H., Tu H., Li S. (2024). Innervation of nociceptor neurons in the spleen promotes germinal center responses and humoral immunity. Cell.

[10] Yu J., Xiao K., Chen X., Deng L., Zhang L., Li Y., Gao A., Gao J., Wu C., Yang X. (2022). Neuron-derived neuropeptide Y regulates splenic immune responses. Neuron.

[11] Motyl G., Krupka W.M., Maslinska M. (2024). The problem of residual pain in the evaluation of rheumatoid arthritis activity. Rheumatology.

[12] Michaud K., Pope J., van de Laar M., Curtis J.R., Kannowski C., Mitchell S., Bell J., Workman J., Paik J., Cardoso A. (2021). Systematic review of the literature on residual symptoms and unmet needs in patients with rheumatoid arthritis. Arthritis Care Res..

[13] Lawrence J.M., Schardien K., Wigdahl B., Nonnemacher M.R. (2023). Roles of neuropathology-associated reactive astrocytes: A systematic review. Acta Neuropathol. Commun..

[14] Amanollahi M., Jameie M., Heidari A., Rezaei N. (2023). The dialogue between neuroinflammation and adult neurogenesis: Mechanisms involved and alterations in neurological diseases. Mol. Neurobiol..

[15] Fisher T.M., Liddelow S.A. (2024). Emerging roles of astrocytes as immune effectors in the central nervous system. Trends Immunol..

[16] Rustenhoven J., Drieu A., Mamuladze T., de Lima K.A., Dykstra T., Wall M., Papadopoulos Z., Kanamori M., Salvador A.F., Baker W. (2021). Functional characterization of dural sinuses as a neuroimmune interface. Cell.

[17] Zhang X., Liu L., Chai Y., Zhang J., Deng Q., Chen X. (2024). Reimagining the meninges from a neuroimmune perspective: A boundary, but not peripheral. J. Neuroinflammation.

[18] Seeliger T., Kramer E., Konen F.F., Zehrfeld N., Beider S., Prenzler N.K., Godecke V., Witte T., Skripuletz T., Ernst D. (2023). Sjogren’s syndrome with and without neurological involvement. J. Neurol..

[19] Brock J., Basu N., Schlachetzki J.C.M., Schett G., McInnes I.B., Cavanagh J. (2023). Immune mechanisms of depression in rheumatoid arthritis. Nat. Rev. Rheumatol..

[20] Carrion-Barbera I., Salman-Monte T.C., Vilchez-Oya F., Monfort J. (2021). Neuropsychiatric involvement in systemic lupus erythematosus: A review. Autoimmun. Rev..

[21] Lu Y.Z., Nayer B., Singh S.K., Alshoubaki Y.K., Yuan E., Park A.J., Maruyama K., Akira S., Martino M.M. (2024). CGRP sensory neurons promote tissue healing through neutrophils and macrophages. Nature.

[22] Atta A.A., Ibrahim W.W., Mohamed A.F., Abdelkader N.F. (2023). Polarization of microglia in nociplasmic pain: Mechanisms and perspectives. Inflammopharmacology.

[23] Goebel A., Krock E., Gentry C., Israel M.R., Jurczak A., Urbina C.M., Sandor K., Vastani N., Maurer M., Cuhadar U. (2021). Passive transfer of fibromyalgia symptoms from patients to mice. J. Clin. Investig..

[24] Midavaine E., Moraes B.C., Benitez J., Rodriguez S.R., Braz J.M., Kochhar N.P., Eckalbar W.L., Tian L., Domingos A.I., Pintar J.E. (2025). Meningeal regulatory T cells inhibit nociception in female mice. Science.

[25] Gonzalez-Rodriguez S., Lorenzo-Herrero S., Sordo-Bahamonde C., Hidalgo A., Gonzalez S., Menendez L., Baamonde A. (2022). Involvement of CD4^+^ and CD8^+^ T lymphocytes in the modulation of CCL4-evoked nociceptive processing in mice. Life Sci..

[26] Taketani Y., Marmalidou A., Dohlman T.H., Singh R.B., Amouzegar A., Chauhan S.K., Chen Y., Dana R. (2020). Restoration of Regulatory T-Cell Function in Dry Eye Disease by Antagonizing Substance P/Neurokinin-1 Receptor. Am. J. Pathol..

[27] Furia A., Liguori R., Donadio V. (2025). Small-Fiber Neuropathy: An Etiology-Oriented Review. Brain Sci..

[28] Daifallah O., Farah A., Dawes J.M. (2023). A role for pathogenic autoantibodies in small fiber neuropathy?. Front. Mol. Neurosci..

[29] Shinkarevsky Fleitman I., Nevo Y., Harel L., Amarilyo G., Dori A., Agmon-Levin N., Kachko L., Zaks Hoffer G., Dabby R., Rabie M. (2020). Small fiber neuropathy associated with autoinflammatory syndromes in children and adolescents. Muscle Nerve.

[30] Gwathmey K.G., Satkowiak K. (2021). Peripheral nervous system manifestations of rheumatologic diseases. J. Neurol. Sci..

[31] Zhao Y., Gan L., Ren L., Lin Y., Ma C., Lin X. (2022). Factors influencing blood-brain barrier permeability. Brain Res..

[32] Candelario-Jalil E., Dijkhuizen R.M., Magnus T. (2022). Neuroinflammation, Stroke, Blood-Brain Barrier Dysfunction, and Imaging Modalities. Stroke.

[33] Galea I. (2021). The blood-brain barrier in systemic infection and inflammation. Cell. Mol. Immunol..

[34] Fitzcharles M.A., Cohen S.P., Clauw D.J., Littlejohn G., Usui C., Hauser W. (2021). Nociplastic pain: Toward an understanding of prevalent pain conditions. Lancet.

[35] Bjorklund G., Dadar M., Pivina L., Dosa M.D., Semenova Y., Maes M. (2020). Environmental, Neuro-immune, and Neuro-oxidative Stress Interactions in Chronic Fatigue Syndrome. Mol. Neurobiol..

[36] Jolly A.A., Brown R.B., Tozer D.J., Hong Y.T., Fryer T.D., Aigbirhio F.I., O’Brien J.T., Markus H.S. (2024). Is central and systemic inflammation associated with fatigue in small vessel disease of the brain?. Int. J. Stroke.

[37] Al-Hakeim H.K., Al-Rubaye H.T., Almulla A.F., Al-Hadrawi D.S., Maes M. (2023). Chronic Fatigue, Depression and Anxiety Symptoms in Long COVID Are Strongly Predicted by Neuroimmune and Neuro-Oxidative Pathways Which Are Caused by the Inflammation during Acute Infection. J. Clin. Med..

[38] Berntson L., Elfving A., Samuelsson A.G., Oman A., Mobarrez F. (2024). Blood-brain barrier permeability and astrocyte-derived extracellular vesicles in children with juvenile idiopathic arthritis: A cross-sectional study. Pediatr. Rheumatol..

[39] Matsushita T., Otani K., Yoshiga M., Hirano M., Noda K., Kurosaka D. (2023). Inhibitory effect of baricitinib on microglia and STAT3 in a weak blood-brain barrier region in a mouse model of rheumatoid arthritis. Rheumatology.

[40] Chimenti M.S., Fonti G.L., Conigliaro P., Triggianese P., Bianciardi E., Coviello M., Lombardozzi G., Tarantino G., Niolu C., Siracusano A. (2021). The burden of depressive disorders in musculoskeletal diseases: Is there an association between mood and inflammation?. Ann. Gen. Psychiatry.

[41] Kwok C.H.T., Kohro Y., Mousseau M., O’Brien M.S., Matyas J.R., McDougall J.J., Trang T. (2021). Role of Primary Afferents in Arthritis Induced Spinal Microglial Reactivity. Front. Immunol..

[42] Schaible H.G., Konig C., Ebersberger A. (2024). Spinal pain processing in arthritis: The (inter)actions of neurons and glia. J. Neurochem..

[43] Borst K., Dumas A.A., Prinz M. (2021). Microglia: Immune and nonimmune functions. Immunity.

[44] Lee H.G., Lee J.H., Flausino L.E., Quintana F.J. (2023). Neuroinflammation: An astrocytic perspective. Sci. Transl. Med..

[45] Makabe K., Okada H., Tachibana N., Ishikura H., Ito N., Tanaka M., Chijimatsu R., Terashima A., Yano F., Asaka M. (2024). Baricitinib ameliorates inflammatory and neuropathic pain in mice with collagen antibody-induced arthritis by modulating IL-6/JAK/STAT3 pathway and CSF-1 expression in dorsal root ganglion neurons. Arthritis Res. Ther..

[46] Suss P., Rothe T., Hoffmann A., Schlachetzki J.C.M., Winkler J. (2020). The Joint-Brain Axis: Insights From Rheumatoid Arthritis on the Crosstalk Between Chronic Peripheral Inflammation and the Brain. Front. Immunol..

[47] Nikolopoulos D., Manolakou T., Polissidis A., Filia A., Bertsias G., Koutmani Y., Boumpas D.T. (2023). Activation of microglia in the presence of an intact blood-brain barrier and disruption of hippocampal neurogenesis by IL-6 and IL-18 mediate early diffuse neuropsychiatric lupus. Ann. Rheum. Dis..

[48] Han X., Xu T., Ding C., Wang D., Yao G., Chen H., Fang Q., Hu G., Sun L. (2022). Neuronal NR4A1 deficiency drives complement-coordinated synaptic stripping by microglia in a mouse model of lupus. Signal Transduct. Target. Ther..

[49] Zhou Y., Chen L., Zheng X., Fang Q., Qian Y., Xu T., Liang J., Zhang H., Han X., Sun L. (2024). Microglia orchestrate synaptic and neuronal stripping: Implication in neuropsychiatric lupus. J. Cell. Mol. Med..

[50] Perez-Nievas B.G. (2023). Special astrocytes release glutamate. Nat. Neurosci..

[51] Liu D., Liao P., Li H., Tong S., Wang B., Lu Y., Gao Y., Huang Y., Zhou H., Shi L. (2024). Regulation of blood-brain barrier integrity by Dmp1-expressing astrocytes through mitochondrial transfer. Sci. Adv..

[52] Jackson R.J., Meltzer J.C., Nguyen H., Commins C., Bennett R.E., Hudry E., Hyman B.T. (2022). Astrocyte-derived APOE4 leads to blood-brain barrier impairment. Brain.

[53] Hanani M. (2024). Satellite glial cells in human diseases. Cells.

[54] Fang S., Wu Z., Guo Y., Zhu W., Wan C., Yuan N., Chen J., Hao W., Mo X., Guo X. (2023). Roles of microglia in adult hippocampal neurogenesis in depression and their therapeutics. Front. Immunol..

[55] Grubic Kezele T., Omrcen H., Baticic L., Sucurovic S., Zoricic Cvek S. (2024). Joint Inflammation Correlates with Joint GPR30 Expression in Males and Hippocampal GPR30 Expression in Females in a Rat Model of Rheumatoid Arthritis. Int. J. Mol. Sci..

[56] Nguyen A.T., Kim H.K. (2023). Recent Developments in PET and SPECT Radiotracers as Radiopharmaceuticals for Hypoxia Tumors. Pharmaceutics.

[57] Alshelh Z., Brusaferri L., Saha A., Morrissey E., Knight P., Kim M., Zhang Y., Hooker J.M., Albrecht D., Torrado-Carvajal A. (2022). Neuroimmune signatures in chronic low back pain subtypes. Brain.

[58] Fukui S., Winkelmayer W.C., Tedeschi S.K., Marrugo J., Guan H., Harrold L., Litman H.J., Shinozaki T., Solomon D.H. (2025). Disease activity of rheumatoid arthritis and kidney function decline: A large prospective registry study. Ann. Rheum. Dis..

[59] Wang Y., Coughlin J.M., Ma S., Endres C.J., Kassiou M., Sawa A., Dannals R.F., Petri M., Pomper M.G. (2017). Neuroimaging of translocator protein in patients with systemic lupus erythematosus: A pilot study using [^11^C]DPA-713 positron emission tomography. Lupus.

[60] Albrecht D.S., Forsberg A., Sandstrom A., Bergan C., Kadetoff D., Protsenko E., Lampa J., Lee Y.C., Hoglund C.O., Catana C. (2019). Brain glial activation in fibromyalgia—A multi-site positron emission tomography investigation. Brain Behav. Immun..

[61] Geladaris A., Torke S., Saberi D., Alankus Y.B., Streit F., Zechel S., Stadelmann-Nessler C., Fischer A., Boschert U., Hausler D. (2024). BTK inhibition limits CNS inflammation perpetuated by microglia and promotes myelin repair. Acta Neuropathol..

[62] Liang Y., Kang X., Zhang H., Xu H., Wu X. (2023). Knockdown and inhibition of hippocampal GPR17 attenuate lipopolysaccharide-induced cognitive impairment in mice. J. Neuroinflammation.

[63] Guo X., Nakamura K., Kohyama K., Harada C., Behanna H.A., Watterson D.M., Matsumoto Y., Harada T. (2007). Inhibition of glial cell activation improves the severity of experimental autoimmune encephalomyelitis. Neurosci. Res..

[64] Devigili G., Cazzato D., Lauria G. (2020). Clinical diagnosis and management of small fiber neuropathy: An update on best practices. Expert Rev. Neurother..

[65] Kool D., Hoeijmakers J.G., Waxman S.G., Faber C.G. (2024). Small fiber neuropathy. Int. Rev. Neurobiol..

[66] Galosi E., Pirone C., Ceccarelli F., Esposito N., Falco P., Leopizzi M., Di Maio V., Tramontana L., De Stefano G., Di Pietro G. (2024). Clinical, histologic, and immunologic signatures of Small Fiber Neuropathy in Systemic Lupus Erythematosus. J. Peripher. Nerv. Syst..

[67] Seeliger T., Dreyer H.N., Siemer J.M., Bonig L., Gingele S., Dohrn M.F., Prenzler N., Ernst D., Witte T., Skripuletz T. (2023). Clinical and paraclinical features of small-fiber neuropathy in Sjogren’s syndrome. J. Neurol..

[68] Birnbaum J. (2017). Small-fiber neuropathy presenting in the antecedent period of undifferentiated arthritis before rheumatoid arthritis. Neurol. Clin. Pract..

[69] Gavrilova N., Starshinova A., Zinchenko Y., Kudlay D., Shapkina V., Malkova A., Belyaeva E., Pavlova M., Yablonskiy P., Shoenfeld Y. (2021). Small Fiber Neuropathy in Sarcoidosis. Pathophysiology.

[70] Saperstein D.S. (2020). Small fiber neuropathy. Neurol. Clin..

[71] Birnbaum J., Lalji A., Saed A., Baer A.N. (2019). Biopsy-Proven Small-Fiber Neuropathy in Primary Sjogren’s Syndrome: Neuropathic pain characteristics, autoantibody findings, and histopathologic features. Arthritis Care Res..

[72] Liampas A., Parperis K., Erotocritou M.F., Nteveros A., Papadopoulou M., Moschovos C., Akil M., Coaccioli S., Hadjigeorgiou G.M., Hadjivassiliou M. (2023). Primary peripheral neuropathy related to Sjogren’s syndrome: A systematic review and meta-analysis. Eur. J. Neurol..

[73] Zeidman L.A., Levine T., Cangelosi J. (2024). Small-Vessel Vasculitis or Perifolliculitis in Small-Fiber Neuropathy with TS-HDS, FGFR-3, or Plexin D1 Antibodies. J. Clin. Neuromuscul. Dis..

[74] Kyle K., Hutto S.K., Reda H., Zonozi R., Farhad K., Jeyabalan A., Chwalisz B.K. (2023). Small fiber neuropathy associated with ANCA positivity: A case series and brief literature review. Neurol. Sci..

[75] Chan A.C.Y., Siah K.T.H. (2024). Can Small Fiber Neuropathy Explain the Overlap Gastrointestinal and Non-gastrointestinal Symptoms in Some Irritable Bowel Syndrome Patients?. J. Neurogastroenterol. Motil..

[76] Azcue N., Del Pino R., Acera M., Fernandez-Valle T., Ayo-Mentxakatorre N., Perez-Concha T., Murueta-Goyena A., Lafuente J.V., Prada A., Lopez de Munain A. (2023). Dysautonomia and small-fiber neuropathy in the post-COVID condition and chronic fatigue syndrome. J. Transl. Med..

[77] Moak J.P., Ramwell C.B., Gordish-Dressman H., Sule S.D., Bettini E. (2024). Small-fiber neuropathy in children, adolescents, and young adults with chronic orthostatic intolerance and tachycardia syndrome postural orthostatic tachycardia: A retrospective study. Auton. Neurosci..

[78] Dumolard A., Lefaucheur J.P., Hodaj E., Liateni Z., Payen J.F., Hodaj H. (2023). Central Sensitization and Small-fiber Neuropathy Are Associated in Patients with Fibromyalgia. Clin. J. Pain.

[79] de Tommaso M., Vecchio E., Nolano M. (2022). The fibromyalgia puzzle between central sensitization syndrome and small fiber neuropathy: A narrative review on neurophysiological and morphological evidence. Neurol. Sci..

[80] Volcheck M.M., Graham S.M., Fleming K.C., Mohabbat A.B., Luedtke C.A. (2023). Central sensitization, chronic pain, and other symptoms: Better understanding, better management. Clevel. Clin. J. Med..

[81] Lepri B., Romani D., Storari L., Barbari V. (2023). Effectiveness of pain neuroscience training in patients with chronic musculoskeletal pain and central sensitization: A Systematic Review. Int. J. Environ. Res. Public Health.

[82] Trouvin A.P., Attal N., Perrot S. (2022). Evaluation of central sensitization with quantitative sensory testing in inflammatory rheumatic diseases: A systematic review. Jt. Bone Spine.

[83] Dougados M., Perrot S. (2017). Fibromyalgia and central sensitization in chronic inflammatory joint disease. Jt. Bone Spine.

[84] Guler M.A., Celik O.F., Ayhan F.F. (2020). The important role of central sensitization in chronic musculoskeletal pain observed in various rheumatic diseases. Clin. Rheumatol..

[85] Kosek E., Cohen M., Baron R., Gebhart G.F., Mico J.A., Rice A.S.C., Rief W., Sluka A.K. (2016). Do we need a third mechanistic descriptor for chronic pain states?. Pain.

[86] Jensen T.S., Finnerup N.B. (2014). Allodynia and hyperalgesia in neuropathic pain: Clinical manifestations and mechanisms. Lancet Neurol..

[87] Melvin B., Wright R., McNally A., Elmofty D. (2025). Allodynia: A Review Article. Curr. Pain Headache Rep..

[88] Sarzi-Puttini P., Giorgi V., Marotto D., Atzeni F. (2020). Fibromyalgia: An update on clinical features, etiopathogenesis, and treatment. Nat. Rev. Rheumatol..

[89] Fitzcharles M.A., Perrot S., Hauser W. (2018). Comorbid fibromyalgia: A qualitative review of prevalence and significance. Eur. J. Pain.

[90] Zhao S.S., Duffield S.J., Goodson N.J. (2019). The prevalence and impact of comorbid fibromyalgia in inflammatory arthritis. Best Pract. Res. Clin. Rheumatol..

[91] Coskun Benlidayi I. (2020). Fibromyalgia interferes with disease activity and response to biological therapy in inflammatory rheumatic diseases. Rheumatol. Int..

[92] Jones G.T., Mallawaarachchi B., Shim J., Lock J., Macfarlane G.J. (2020). The prevalence of fibromyalgia in axial spondyloarthritis. Rheumatol. Int..

[93] Niu Y., Zeng X., Qin G., Zhang D., Zhou J., Chen L. (2021). Downregulation of metabotropic glutamate 5 receptor alleviates central sensitization by activating autophagy through inhibition of mTOR pathway in a rat model of chronic migraine. Neurosci. Lett..

[94] Merighi A. (2024). Brain-Derived Neurotrophic Factor, Nociception, and Pain. Biomolecules.

[95] Nugraha B., Karst M., Engeli S., Gutenbrunner C. (2012). Brain-derived neurotrophic factor and exercise in fibromyalgia syndrome patients: A mini review. Rheumatol. Int..

[96] Souza Monteiro de Araujo D., Nassini R., Geppetti P., De Logu F. (2020). TRPA1 as a therapeutic target for nociceptive pain. Expert Opin. Ther. Targets.

[97] De Preter C.C., Heinricher M.M. (2024). The ‘in’s and out’s’ of descending pain modulation from the rostral ventromedial medulla. Trends Neurosci..

[98] Gao Z.R., Chen W.Z., Liu M.Z., Chen X.J., Wan L., Zhang X.Y., Yuan L., Lin J.K., Wang M., Zhou L. (2019). Tac1-expressing neurons in the periaqueductal gray facilitate the pruritus-gratate cycle through down-regulation. Neuron.

[99] Pinto-Ribeiro F., Amorim D., David-Pereira A., Monteiro A.M., Costa P., Pertovaara A., Almeida A. (2013). Pronoception from the dorsomedial nucleus of the hypothalamus is mediated by the rostral ventromedial medulla in healthy controls but is absent in arthritic animals. Brain Res. Bull..

[100] Kandemirli S.G., Bathla G. (2021). Neuroimaging findings in rheumatologic disorders. J. Neurol. Sci..

[101] Vassilaki M., Crowson C.S., Davis Iii J.M., Duong S.Q., Jones D.T., Nguyen A., Mielke M.M., Vemuri P., Myasoedova E. (2022). Rheumatoid Arthritis, Cognitive Impairment, and Neuroimaging Biomarkers: Results from the Mayo Clinic Study of Aging. J. Alzheimer’s Dis..

[102] Taylor P.C. (2023). Joint pain and beyond: The challenge of rheumatoid arthritis. Lancet Rheumatol..

[103] de la Coba P., Montoro C.I., Reyes Del Paso G.A., Galvez-Sanchez C.M. (2022). Algometry for the assessment of central pain sensitization in fibromyalgia patients: A systematic review. Ann. Med..

[104] Kumthekar A., Ashrafi M., Deodhar A. (2023). Difficult-to-treat psoriatic arthritis: How to manage it?. Clin. Rheumatol..

[105] Currado D., Saracino F., Ruscitti P., Marino A., Pantano I., Vomero M., Berardicurti O., Pavlych V., Di Vico C., Caso F. (2024). Pain catastrophizing negatively affects drug retention rate in patients with psoriatic arthritis and axial spondyloarthritis: Results of a 2-year multicenter GIRRCS (Italian Research Group in Clinical Rheumatology) study. Arthritis Res. Ther..

[106] Curtis J.R., Herrem C., Ndlovu N., O’Brien C., Yazici Y. (2017). A somatization comorbidity phenotype affects response to therapy in rheumatoid arthritis: Post-hoc results from the phase 4 PREDICT study of certolizumab pegol. Arthritis Res. Ther..

[107] Cooper T.E., Derry S., Wiffen P.J., Moore R.A. (2017). Gabapentin for fibromyalgia pain in adults. Cochrane Database Syst. Rev..

[108] VanderWeide L.A., Smith S.M., Trinkley K.E. (2015). A systematic review of the efficacy of venlafaxine for the treatment of fibromyalgia. J. Clin. Pharm. Ther..

[109] Macfarlane G.J., Kronisch C., Dean L.E., Atzeni F., Hauser W., Fluss E., Choy E., Kosek E., Amris K., Branco J. (2017). EULAR revised recommendations for the management of fibromyalgia. Ann. Rheum. Dis..

[110] Serrat M., Sanabria-Mazo J.P., Almirall M., Muste M., Feliu-Soler A., Mendez-Ulrich J.L., Sanz A., Luciano J.V. (2021). Effectiveness of a Multicomponent Treatment Based on Pain Neuroscience Education, Therapeutic Exercise, Cognitive Behavioral Therapy, and Mindfulness in Patients with Fibromyalgia (FIBROWALK Study): A Randomized Controlled Trial. Phys. Ther..

[111] Ambrose K.R., Golightly Y.M. (2015). Exercise as a nonpharmacologic treatment of chronic pain: Why and when. Best Pract. Res. Clin. Rheumatol..

[112] Wehrwein E.A., Orer H.S., Barman S.M. (2016). Overview of the Anatomy, Physiology, and Pharmacology of the Autonomic Nervous System. Compr. Physiol..

[113] Davies K., Ng W.F. (2021). Autonomic Nervous System Dysfunction in Primary Sjogren’s Syndrome. Front. Immunol..

[114] Bortoluzzi A., Silvagni E., Furini F., Piga M., Govoni M. (2019). Peripheral nervous system involvement in systemic lupus erythematosus: A review of the evidence. Clin. Exp. Rheumatol..

[115] Bellocchi C., Carandina A., Montinaro B., Targetti E., Furlan L., Rodrigues G.D., Tobaldini E., Montano N. (2022). The Interplay between Autonomic Nervous System and Inflammation across Systemic Autoimmune Diseases. Int. J. Mol. Sci..

[116] Tumiati B., Perazzoli F., Negro A., Pantaleoni M., Regolisti G. (2000). Heart rate variability in patients with Sjogren’s syndrome. Clin. Rheumatol..

[117] Erelund S., Sodergren A., Wiklund U., Sundstrom N. (2023). Heart rate variability and cardiovascular risk factors in patients with rheumatoid arthritis: A longitudinal study. Auton. Neurosci..

[118] Rovsing C., Rovsing H., Liboriussen C.H., Jensen M.K., Andersen S.S., Andersen S.S., Kristensen S., Jochumsen M. (2021). Deep breathing increases heart rate variability in patients with rheumatoid arthritis and systemic lupus erythematosus. J. Clin. Rheumatol..

[119] Stojanovich L. (2009). Autonomic dysfunction in autoimmune rheumatic disease. Autoimmun. Rev..

[120] Tracey K.J. (2002). The inflammatory reflex. Nature.

[121] Occhinegro A., McAllen R.M., McKinley M.J., Martelli D. (2023). Acute inhibition of sympathetic nerve-mediated inflammation: The inflammatory reflex. Neuroimmunomodulation.

[122] Occhinegro A., Wong C.Y., Chua B.Y., Jackson D.C., McKinley M.J., McAllen R.M., Martelli D. (2021). Endogenous inflammatory reflex inhibits the inflammatory response to different immune challenges in mice. Brain Behav. Immun..

[123] Yang H., George S.J., Thompson D.A., Silverman H.A., Tsaava T., Tynan A., Pavlov V.A., Chang E.H., Andersson U., Brines M. (2022). Famotidine activates the vagus nerve inflammatory reflex to attenuate cytokine storm. Mol. Med..

[124] Oyama J., Node K. (2014). Sympathetic nerve activity and endothelial function. Hypertens. Res..

[125] Rasmussen S.E., Pfeiffer-Jensen M., Drewes A.M., Farmer A.D., Deleuran B.W., Stengaard-Pedersen K., Brock B., Brock C. (2018). Vagal influences in rheumatoid arthritis. Scand. J. Rheumatol..

[126] Tyagi S., Higerd-Rusli G.P., Ghovanloo M.R., Dib-Hajj F., Zhao P., Liu S., Kim D.H., Shim J.S., Park K.S., Waxman S.G. (2024). Compartment-specific regulation of Na(V)1.7 in sensory neurons after acute exposure to TNF-alpha. Cell Rep..

[127] Wheeler M.A., Heffner D.L., Kim S., Espy S.M., Spano A.J., Cleland C.L., Deppmann C.D. (2014). TNF-alpha/TNFR1 signaling is required for primary nociceptor development and function. Neuron.

[128] Zhou Y.Q., Liu Z., Liu Z.H., Chen S.P., Li M., Shahveranov A., Ye D.W., Tian Y.K. (2016). Interleukin-6: An emerging regulator of pathological pain. J. Neuroinflammation.

[129] Choy E.H.S., Calabrese L.H. (2018). Neuroendocrine and neurophysiologic effects of interleukin-6 in rheumatoid arthritis. Rheumatology.

[130] Wanigatunga A.A., Varadhan R., Simonsick E.M., Carlson O.D., Studenski S., Ferrucci L., Schrack J.A. (2019). Longitudinal Relationship Between Interleukin-6 and Perceived Fatigability Among Well-Functioning Adults in Mid-to-Late Life. J. Gerontol. A Biol. Sci. Med. Sci..

[131] Wang J., Wu T., Zhao Y., Mao L., Ding J., Wang X. (2024). IL-17A exacerbates blood-brain barrier disruption through activation of Src signaling in epileptic mice. Neurobiol..

[132] Khan A.W., Farooq M., Hwang M.J., Haseeb M., Choi S. (2023). Autoimmune neuroinflammatory diseases: Role of interleukins. Int. J. Mol. Sci..

[133] Brigas H.C., Ribeiro M., Coelho J.E., Gomes R., Gomez-Murcia V., Carvalho K., Faivre E., Costa-Pereira S., Darrigues J., de Almeida A.A. (2021). IL-17 triggers the onset of cognitive and synaptic deficits in the early stages of Alzheimer’s disease. Cell Rep..

[134] Schou W.S., Ashina S., Amin F.M., Goadsby P.J., Ashina M. (2017). Calcitonin gene-related peptide and pain: A systematic review. J. Headache Pain.

[135] Iyengar S., Ossipov M.H., Johnson K.W. (2017). The role of calcitonin gene-related peptide in peripheral and central pain mechanisms including migraine. Pain.

[136] Ding W., Stohl L.L., Xu L., Zhou X.K., Manni M., Wagner J.A., Granstein R.D. (2016). Calcitonin Gene-Related Peptide-Exposed Endothelial Cells Bias Antigen Presentation to CD4+ T Cells toward a Th17 Response. J. Immunol..

[137] Mikami N., Sueda K., Ogitani Y., Otani I., Takatsuji M., Wada Y., Watanabe K., Yoshikawa R., Nishioka S., Hashimoto N. (2014). Calcitonin gene-related peptide regulates type IV hypersensitivity through dendritic cell functions. PLoS ONE.

[138] Schank J.R., Heilig M. (2017). Substance P and the neurokinin-1 receptor: The novel CRF. Int. Rev. Neurobiol..

[139] Puts S., Liberman K., Leysen L., Forti L., Muyldermans E., Vaes P., Nijs J., Beckwee D., Bautmans I. (2023). Effects of exercise on inflammatory markers and brain-derived neurotrophic factor in patients with knee osteoarthritis. A systematic review with meta-analysis. Exerc. Immunol. Rev..

[140] Tanaka T., Okuda H., Isonishi A., Terada Y., Kitabatake M., Shinjo T., Nishimura K., Takemura S., Furue H., Ito T. (2023). Cutaneous macrophages regulate pain sensitivity by modulating the amount of tissue NGF through an SNX25-Nrf2 pathway. Nat. Immunol..

[141] Zaninelli T.H., Factors V., Heintz O.K., Wright K.R., Bennallack P.R., Sim D., Bukhari H., Terry K.L., Vitonis A.F., Missmer S.A. (2024). Targeting NGF but not VEGFR1 or BDNF signaling reduces endometriosis-associated pain in mice. J. Adv. Res..

[142] Patel F., Hess D.K., Maher D.P. (2020). Antibodies against nerve growth factor for the treatment of low back pain. Expert Rev. Clin. Pharmacol..

[143] Sun W., Ma M., Yu H., Yu H. (2018). Inhibition of specific lncRNA X-inactivated transcript ameliorates inflammatory pain by suppressing satellite glial cell activation and inflammation by acting as a sponge of miR-146a to inhibit Na(v)1,7. J. Cell. Biochem..

[144] Zhang Y., Chen Q., Nai Y., Cao C. (2020). Suppression of miR-155 attenuates neuropathic pain by inducing a switch from M1 to M2 in microglia. Folia Neuropathol..

[145] Zhang Y., Liu H.L., An L.J., Li L., Wei M., Ge D.J., Su Z. (2019). miR-124-3p attenuates neuropathic pain induced by chronic sciatic nerve injury in rats through targeting EZH2. J. Cell. Biochem..

[146] Kumar V. (2019). Toll-like receptors in the pathogenesis of neuroinflammation. J. Neuroimmunol..

[147] Rodriguez-Palma E.J., Huerta de la Cruz S., Islas-Espinoza A.M., Castaneda-Corral G., Granados-Soto V., Khanna R. (2024). Nociplastic pain mechanisms and toll-like receptors as promising targets for its management. Pain.

[148] Hu S.Q., Hu J.L., Zou F.L., Liu J.P., Luo H.L., Hu D.X., Wu L.D., Zhang W.J. (2022). P2X7 receptor in inflammation and pain. Brain Res. Bull..

[149] Aminin D., Illes P. (2021). Purinergic Signaling in Neuroinflammation. Int. J. Mol. Sci..

[150] Fadda G., Flanagan E.P., Cacciaguerra L., Jitprapaikulsan J., Solla P., Zara P., Sechi E. (2022). Myelitis features and outcomes in CNS demyelinating disorders: Comparison between multiple sclerosis, MOGAD, and AQP4-IgG-positive NMOSD. Front. Neurol..

[151] Liu Y., Tu Z., Zhang X., Du K., Xie Z., Lin Z. (2022). Pathogenesis and treatment of neuropsychiatric systemic lupus erythematosus: A review. Front. Cell Dev. Biol..

[152] Sletten D.M., Suarez G.A., Low P.A., Mandrekar J., Singer W. (2012). COMPASS 31: A refined and abbreviated Composite Autonomic Symptom Score. Mayo Clin. Proc..

[153] Puri B.K., Lee G.S. (2022). Clinical assessment of autonomic function in fibromyalgia using the Refined and Abbreviated Composite Autonomic Symptom Score (COMPASS 31): A case-control study. Rev. Recent Clin. Trials.

[154] Pindi Sala T., Villedieu M., Damian L., Crave J.C., Pautot V., Stojanovich L., Tervaert J.W.C., Cherin P., Belizna C. (2020). Long-term efficacy of immunoglobulins in Sjogren’s syndrome-related small fiber neuropathy. J. Neurol..

[155] Giordano F., Zicca A., Barba C., Guerrini R., Parent L. (2017). Vagus nerve stimulation: Surgical technique of implantation and revision and related morbidity. Epilepsia.

[156] Kudrina I., Shir Y., Fitzcharles M.A. (2015). Multidisciplinary treatment of rheumatic pain. Best Pract. Res. Clin. Rheumatol..

